# Does clinical supervision of healthcare professionals improve effectiveness of care and patient experience? A systematic review

**DOI:** 10.1186/s12913-017-2739-5

**Published:** 2017-11-28

**Authors:** David A. Snowdon, Sandra G. Leggat, Nicholas F. Taylor

**Affiliations:** 1 School of Allied Health, La Trobe University, Bundoora, VIC 3086 Australia; 2 School of Public Health, La Trobe University, Bundoora, VIC 3086 Australia; 3 Allied Health Clinical Research Office Eastern Health, Level 2/5 Arnold Street, Box Hill, VIC 3128 Australia

**Keywords:** Clinical supervision, Effectiveness of care, Patient experience, Quality of care

## Abstract

**Background:**

To ensure quality of care delivery clinical supervision has been implemented in health services. While clinical supervision of health professionals has been shown to improve patient safety, its effect on other dimensions of quality of care is unknown. The purpose of this systematic review is to determine whether clinical supervision of health professionals improves effectiveness of care and patient experience.

**Methods:**

Databases MEDLINE, PsychINFO, CINAHL, EMBASE and AMED were searched from earliest date available. Additional studies were identified by searching of reference lists and citation tracking. Two reviewers independently applied inclusion and exclusion criteria. The quality of each study was rated using the Medical Education Research Study Quality Instrument. Data were extracted on effectiveness of care (process of care and patient health outcomes) and patient experience.

**Results:**

Seventeen studies across multiple health professions (medical (*n* = 4), nursing (*n* = 7), allied health (*n* = 2) and combination of nursing, medical and/or allied health (*n* = 4)) met the inclusion criteria. The clinical heterogeneity of the included studies precluded meta-analysis. Twelve of 14 studies investigating 38,483 episodes of care found that clinical supervision improved the process of care. This effect was most predominant in cardiopulmonary resuscitation and African health settings. Three of six studies investigating 1756 patients found that clinical supervision improved patient health outcomes, namely neurological recovery post cardiopulmonary resuscitation (*n* = 1) and psychological symptom severity (*n* = 2). None of three studies investigating 1856 patients found that clinical supervision had an effect on patient experience.

**Conclusions:**

Clinical supervision of health professionals is associated with effectiveness of care. The review found significant improvement in the process of care that may improve compliance with processes that are associated with enhanced patient health outcomes. While few studies found a direct effect on patient health outcomes, when provided to mental health professionals clinical supervision may be associated with a reduction in psychological symptoms of patients diagnosed with a mental illness. There was no association found between clinical supervision and the patient experience.

**Review Registration:**

CRD42015029643.

**Electronic supplementary material:**

The online version of this article (10.1186/s12913-017-2739-5) contains supplementary material, which is available to authorized users.

## Background

### Rationale/objectives

As part of the process of ensuring quality of care, clinical supervision has been widely implemented throughout health services [1–3]. Many studies have conceptualised clinical supervision of health professionals as senior clinicians overseeing and guiding the practice of less experienced clinicians [1, 2, 4]. Therefore, for the purpose of this systematic review clinical supervision was defined as ‘the provision of guidance of clinical practice for qualified health professionals by a more experienced health professional’ [1, 2, 4]. Clinical supervision is a professional development activity where the less experienced clinician can utilise the knowledge and experience of their supervisor, to address any gaps in knowledge or skill set and thereby improve their own clinical performance and patient quality of care [1, 2, 4].

Contemporary definitions of quality of care include three components: care that is clinically effective, care that is safe and care that provides a positive experience for patients [5]. Care that is clinically effective refers to providing beneficial interventions at the right time to the right patients, and includes measures of process and patient health outcomes; care that is safe reduces and controls the risk of patient harm; and the patient should be the focus of health care intervention to ensure that their experience is tailored to their needs [6].

When investigating the effectiveness of a professional development activity, such as clinical supervision, it is important that the effect on quality of care is measured [7]. The effects of clinical supervision of health professionals on patient safety have been established in a recent systematic review. In this systematic review, which investigated the effects of experienced health professionals guiding the practice of less experienced professionals, the authors concluded that clinical supervision is associated with a reduced risk of adverse patient outcomes (e.g. mortality) during high risk, invasive procedures such as surgery [8]. The authors of another systematic concluded that clinical supervision of medical residents may be beneficial at improving residents’ clinical skills, competency and adherence with protocols, and reducing patient complications [9]. However, patient health outcomes and patient experience were not evaluated, and the results cannot be generalised to supervision of all health professionals. Therefore, little is known about the effect of clinical supervision on effectiveness of care and patient satisfaction for all health professionals, including medical, nursing and allied health professionals.

The main aim of this systematic review was to investigate the effect of clinical supervision of health professionals on the effectiveness of patient care and the patient experience.

## Methods

### Protocol and registration

This systematic review has been reported with reference to the Preferred Reporting Items for Systematic Reviews and Meta-Analyses (PRISMA) guidelines for reporting of systematic reviews and meta-analyses [10] and has been registered prospectively in the PROSPERO database (registration number: CRD42015029643).

### Eligibility criteria

To be eligible, studies had to meet the following criteria: (1) investigated clinical supervision of qualified, registered or postgraduate trainee health professionals; (2) measured effectiveness of care utilising either measures of process (e.g. compliance with practice guidelines) or patient health outcomes (e.g. body structure, body function, activity, participation and quality of life measures) OR investigated patient experience with healthcare services (3) investigated a model of clinical supervision where the supervisor had more experience/expertise than the supervisee and involved supervision of clinical practice; (4) included a control group or historical comparison of health professionals who did not receive supervision or received less supervision; (5) were written in English.

Studies were ineligible if they met any of the following criteria: (1) investigated the effects of undergraduate student or entry level clinical supervision; (2) investigated the effect of clinical supervision on the performance of the clinical supervisor; (3) measured the effect of clinical supervision utilising only environmental outcomes (e.g. use of social supports) or patient safety outcomes (4) measured quality of care utilising therapist self-reported perception (5) investigated supervision of simulated patient care scenarios; (6) investigated the effects of clinical supervision of non-health care professionals; (7) investigated a peer supervision model. Inclusion/exclusion criteria ensured that studies fulfilled the definition of clinical supervision, utilised in a previous systematic review, as ‘the provision of guidance of clinical practice for qualified health professionals by a more experienced health professional’ [1, 2, 4, 8], and that outcomes provided data to address the review aims.

### Information sources

From the earliest available date until 11th April 2015 the electronic databases MEDLINE, PsychINFO, CINAHL, EMBASE and AMED were searched. Database searching was supplemented by hand searching reference lists of included studies and citation tracking using Google scholar.

### Search

The concept of intervention was searched using the following key words: supervis*, mentor*, debrief* and reflective practice. The concept of outcomes was searched using the following key words: patient outcomes, clinical outcomes, client outcomes, patient care, quality of care, patient experience, adherence and compliance. Key words and synonyms for each concept were combined with the ‘OR’ operator. The concepts of supervision and outcome were combined with the ‘AND’ operator. An example search strategy for the Medline database is provided in an additional file (see Additional file 1).

### Study selection

One reviewer (DS) screened the articles by title and abstract utilising the eligibility criteria. Another reviewer (NT) screened the first 200 articles by title and abstract to check there was consistency in the application of the eligibility criteria. Agreement between the reviewers was reported with the kappa statistic (κ) and if κ < 0.75, the second reviewer screened a further 200 articles until acceptable agreement could be reached. Full text copies of articles that were not definitely excluded on title and abstract were retrieved for detailed examination. The two reviewers then independently reapplied the eligibility criteria on all full text copies. Uncertain cases were discussed by the reviewers to achieve consensus.

### Data collection process

Pre-designed spread sheets were used to extract data on participants, healthcare interventions, supervision interventions and outcomes. The primary outcomes reflective of the effectiveness of care and patient experience dimensions of quality of care [5] were patient health outcomes, process measures including therapist compliance with guidelines/protocols relating to patient management, and measures of patient experience [11–15]. Patient health outcomes of interest were body structure, body function, activity, participation and health-related quality of life measures but not environmental outcomes, such as social supports, or patient safety outcomes, such as mortality, adverse event/complications, failure to treat and re-admission.

Supervision interventions were classified as: direct supervision of clinical practice, debriefing/reflective practice, and a combination of both direct supervision and debriefing/reflective practice [8]. Direct supervision refers to supervision of clinical practice where the supervisor is personally present, either face-to-face or using a communication device, during the occasion of service and has the potential to immediately influence patient care [16, 17]. Debriefing/reflective practice refers to supervision of clinical practice that occurs after patient contact and requires the supervisee to critically reflect on their clinical performance prior to any alteration in patient care [18].

Supervision was also described in terms of the frequency of supervision and the clinical practice that was supervised. Supervised clinical practice was classified as: supervision of general practice; supervision of a procedure or treatment technique; or supervision of a specified area of clinical practice.

### Methodological quality in individual studies

Studies were critically appraised for methodological quality using the Medical Education Research Study Quality Instrument (MERSQI) [19]. The MERSQI is a 10-item quality assessment tool that reflects 6 domains of study quality with a score range of 5–18 for total score [19]. A MERSQI score of 11 or higher was interpreted as a study of higher quality [20]. All studies were independently assessed by two reviewers (DS and NT). Inter-rater agreement was recorded and expressed with κ. Any disagreements between reviewers were resolved through discussion.

### Synthesis of results

Odds ratios (OR) of dichotomous events and standardised mean differences (SMD) for continuous measures were calculated from measures of compliance with process and patient outcome data. Where studies were sufficiently homogenous in terms of participants, supervision interventions, therapeutic interventions and outcomes, a meta-analysis of dichotomous and/or continuous outcomes was planned utilising the inverse variance method and random-effects model [21]. If combining data were not appropriate due to clinical heterogeneity results were synthesised descriptively.

## Results

### Study selection

**Fig. 1:**
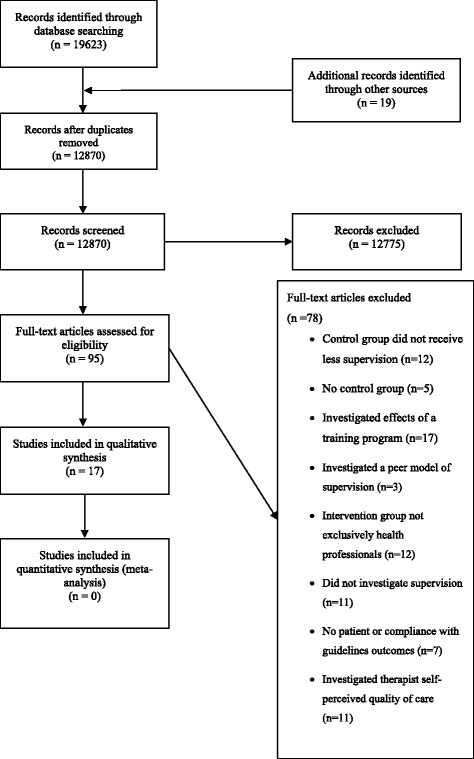
Article selection process

The database search yielded 19,623 records. Ninety-five articles were retrieved for full text review following application of the eligibility criteria to title and abstract. Agreement between reviewers for screening the first 200 articles was very good (κ = 0.91, 95%CI 0.73 to 1.00). Fourteen studies fulfilled the inclusion criteria when applied to full texts. Nineteen records were identified for full text review from reference lists of included articles and citation tracking. Three of these articles fulfilled the inclusion criteria, hence the final yield was 17 studies (Fig. 1). Agreement between reviewers for screening full text articles was very good (κ = 0.88, 95%CI 0.74 to 1.00).

### Study characteristics & methodological quality within studies

Of the 17 studies included in this systematic review: six investigated patient health outcomes of 1746 patients [22–27]; 14 investigated process of care measures for 38,483 episodes of patient care [24–37]; and three studies investigated the health care experience of 1856 patients [25, 35, 38]. Five studies investigated direct supervision [25, 31, 33–35], six studies investigated debriefing/reflective practice [22–24, 26–28, 38] and five studies investigated a combination of direct supervision and debriefing/reflective practice [29, 30, 32, 36, 37]. Four studies investigated clinical supervision of medical professionals [25, 28, 34, 35], seven studies investigated clinical supervision of nursing professionals [23, 29–32, 37, 38], two studies investigated clinical supervision of allied health professionals [22, 24] and three studies investigated supervision of a combination of health professions [26, 27, 33, 36]. Clinical supervision was conducted weekly in three studies [25, 26, 28], fortnightly in two studies [23, 30] and monthly in four studies [29, 32, 33, 37]. Four studies did not report frequency of supervision sessions [34–36, 38] and two studies reported supervision sessions occurring following a clinical event/consultation [24, 27]. The quantity of supervision sessions was reported in two studies with participants receiving 40 h of supervision in one study [31] and 8 sessions in another [22]. Clinical supervision was predominantly provided for a specific area of clinical practice (*n* = 9) [22, 23, 29–34, 37] or a procedure/treatment technique (*n* = 5) [24, 26–28, 36], while clinical supervision of general practice was investigated by fewer studies (*n* = 3) [25, 35, 38].

The mean MERSQI score for included studies was 13.1 (κ = 0.68, 95%CI 0.57 to 0.80), with 16 studies scoring 11 or higher. Three studies utilised a randomised controlled trial design [22, 24, 37], eight studies utilised a single group, pre-test post-test design [27–33, 36], two utilised a retrospective cohort design [34, 35] and four studies utilised a prospective cohort design with either concurrent or historical control [23, 25, 26, 38]. One study utilised both a concurrent (between hospital) and historical (within hospital) control; for this review we analysed the data from the historical control [26]. A table outlining study characteristics is provided in an additional file (see Additional file 2).

### Results of individual studies

Due to clinical heterogeneity no meta-analyses were completed.

### Patient health outcomes

Three of the six patient health outcome studies investigated the effects of reflective supervision of mental health professionals on patient health outcomes [22–24]. Two studies found that patients managed by mental health professionals who participated in reflective practice, had a lower severity of psychological symptoms than patients managed by unsupervised professionals [22, 23]. The third study found that reflective supervision of counsellors had no significant effect on patient substance abuse [24].

The three remaining studies investigated the effect of direct supervision of outpatient medical professionals [25] and reflective supervision of a multi-disciplinary medical emergency response team [26, 27]. Reflective supervision of a multidisciplinary team providing cardiopulmonary resuscitation (CPR) for adult patients with cardiac arrest was not associated with an improved neurologic outcome [26]. In contrast, supervision of a multidisciplinary team providing CPR for paediatric patients was associated with an improved neurologic outcome [27]. Outpatients, presenting with a range of conditions including cardiac, gastrointestinal, pulmonary and renal, managed by directly supervised medical professionals did not have significantly greater health outcomes compared to those managed by unsupervised professionals [25] (Table 1).

**Table 1:** Effect of supervision on patient health outcomes

Study	N	Measure	Method	Results: Supervision vs. Control (SMD > 0 favours supervision) (OR >1 favours supervision)
Bambling et al. 2006 [22]	103	Depression severity	Beck Depression Inventory	Skills Foci Supervision BDI **SMD 0.50 (95%CI 0.02 to 0.99)** Process Foci Supervision BDI **SMD 0.76 (95%CI 0.28 to 1.24)**
Bradshaw et al. 2007 [23]	93	Psychiatric symptoms in individuals experiencing psychotic symptoms	Krawiecka, Goldberg and Vaughan symptom scale (KGV)	KGV Affective Symptoms SMD 0.32 (95%CI −0.10 to 0.73) KGV Positive Symptoms **SMD 0.48 (95%CI 0.06 to 0.90)** KGV Negative Symptoms SMD 0.06 (95%CI −0.36 to 0.47) KGV Total Symptom Score **SMD 0.47 (95%CI 0.06 to 0.89)**
Couper et al. 2015 [26]	746	Neurologic Outcome	Cerebral Performance Category (CPC) Score; analysed dichotomously as good (CPC 1 or 2) OR poor (CPC 3, 4 or 5)	Good Neurologic Outcome OR 1.02 (95%CI 0.70 to 1.48)
Martino et al. 2016 [24]	385	Days of primary substance abuse abstinence	Self-report of substance abuse utilising the substance use calendar	SMD −0.06 (95%CI −0.26 to 0.14)
Pozen et al. 1976 [25]	300	Faculty member ratings of patient outcomes, including patient symptoms function and health status	Medical record review, patient questionnaire and 8-month follow-up assessment	N/S^a^
Wolfe et al. 2014 [27]	119	Neurologic Outcome	Paediatric Cerebral Performance Category (PCPC) Score; analysed dichotomously as favourable (PCPC score 1–3 OR no change from admission score) or non-favourably (PCPC score 4–6)	Favourable Neurologic Outcome **OR 2.47 (95%CI 1.05 to 5.78)**

bold text, *P* < .05

*N/S* non-significant

^a^insufficient data provided to calculate SMD/OR

### Process of care

Reflective supervision of health professionals significantly improved their performance of CPR [26–28]. There were significant improvements in the quality indicators of compression rate [26–28], compression depth [26–28] flow fraction [26–28], pre-shock pause [26, 28], post-shock pause [26, 28], incomplete release [26], ventilation rate [28] and delivery of appropriate shocks [28]. Additionally, one study found that clinical supervision increased the percentage of CPR attempts that were performed within recommended guidelines [27].

Studies investigating clinical supervision of health professionals delivering care in African health settings, found positive results for improved care processes [29–33]. Compared to historical controls, the introduction of a clinical supervision intervention improved: (1) the delivery of anti-retroviral therapy by medical and nursing professionals [31, 33]; (2) performance of procedures that aim to reduce mother-to-child transmission of HIV [30]; and (3) adherence to nursing care guidelines in the management of antenatal [29], childhood [29, 32] and adult illness [29]. Two studies utilised a direct supervision model [31, 33] and three studies, a combination of direct and reflective supervision [29, 30, 32].

Two studies found that direct supervision of medical residents improved their compliance with guidelines for the management of patients requiring emergency care [34, 35]. Similar results were not found in an outpatient setting, where direct supervision of interns and residents had no impact on the process of care [25]. One cluster randomised controlled trial found that a combination of direct and reflective supervision improved intra-partum and post-partum nursing care in Indian health centres [37]. While another study investigating the effect of combined direct and reflective supervision of medical, nursing and allied health professionals, found improved adherence to acceptability and repeatability criteria for performance of spirometry [36].

A randomised controlled trial found that reflective supervision, with a focus on motivational interviewing techniques, had no effect on counsellor adherence to motivational interviewing strategies when compared to usual practice supervision [24].

Compliance with procedures was assessed by observation of clinical practice in two studies [29, 32], review of audiotape in one study [24], medical record review or analysis of routinely collected data in eight studies [25, 30, 31, 33–37] and defibrillators/monitors with detectors in three studies investigating the quality of CPR [26–28] (Table 2).

**Table 2:** Effect of supervision on process measures

Study	N	Measure	Method	Results: Supervision vs. Control (SMD > 0 favours supervision) (OR >1 favours supervision)
Anatole et al. 2013 [29]	2649	Adherence to national paediatric nursing guidelines Adherence to national adult nursing guidelines Adherence to national antenatal nursing guidelines	Observation of nurse clinical practice by supervisors	Paediatric: **SMD 0.94 (95%CI 0.80 to 1.08)** Adult: **SMD 2.21 (95%CI 1.97 to 2.45)** Antenatal: **SMD 0.50 (95%CI 0.34 to 0.65)**
Claridge et al. 2011 [34]	376	Adherence to protocol for selection of non-operative management in patients with a blunt spleen injury	Medical record and trauma registry review	**OR 3.99 (95%CI 1.94 to 8.19)**
Couper et al. 2015 [26]	746	CPR performance quality metrics: compression rate (no/min); compression depth (mm); flow-fraction (%); incomplete release (%); pre-shock pause (secs), post-shock pause (secs).	Monitor and defibrillator with the capability to detect and record chest compressions and ventilations during resuscitation attempts	Compression Rate **SMD 0.25 (95%CI 0.10 to 0.39)** Compression Depth **SMD 0.60 (95%CI 0.45 to 0.74)** Flow Fraction **SMD 0.41 (95%CI 0.26 to 0.55)** Incomplete Release **SMD 0.16 (95%CI 0.02 to 0.30)** Pre-Shock Pause **SMD 0.81 (95%CI 0.66 to 0.96)** Post-Shock Pause **SMD 0.57 (95%CI 0.42 to 0.71)**
Edelson et al. 2008 [28]	224	CPR performance quality metrics: 5-min compression depth (mm); 5-min compression rate (no/min); 5-min ventilation rate (no/min); 5-min no-flow fraction; pre-shock pause (secs); post-shock pause (secs); and appropriate number of shocks	Monitor and defibrillator with the capability to detect and record chest compressions and ventilations during resuscitation attempts	5-Minute Compression Depth **SMD 0.60 (95%CI 0.33 to 0.87)** 5-Minute Compression Rate **SMD 0.44 (95%CI 0.17 to 0.70)** 5-Minute Ventilation Rate **SMD 0.67 (95%CI 0.40 to 0.94)** 5-Minute No-Flow Fraction **SMD 0.61 (95%CI 0.34 to 0.88)** Post-Shock Pause **Median (IQR) 7.5 (2.8 to 13.1) vs. 16.0 (8.5 to 24.1), P < 0.001** ^a^ Pre-Shock Pause **Median (IQR) 2.4 (1.9 to 3.6) vs. 7.1 (2.7 to 14.8), P < 0.001** ^a^ Appropriate Shocks **OR 2.98 (95%CI 1.51 to 5.88)**
Fatti et al. 2013 [30]	27,458	Adherence to prevention of mother to child HIV transmission guidelines	Analysis of routine clinical data	Adherence to Guidelines **OR 1.41 (95%CI 1.36 to 1.45)**
Green et al. 2014 [31]	160	Adherence to nurse-administered antiretroviral therapy guidelines	Medical record review	Adherence to Guidelines **OR 1.52 (95%CI 1.27 to 1.83)**
Gupta et al. 2016 [36]	384	Adherence to acceptability and repeatability criteria of the American Thoracic Society/European Respiratory Society standards for spirometry	Review of spirometry results/output	Adherence to Guidelines **OR 1.7 (95%CI 1.0 to 3.0)**
Jayanna et al. 2016 [37]	1078	Adherence to intra-partum and post-partum nursing care guidelines	Medical record review	Adherence to Initial Assessment Guidelines **OR 3.6 (95%CI 1.7 to 7.6)** Adherence to Labour Monitoring Guidelines **OR 25.8 (95%CI 9.6 to 69.4)** Adherence to Delivery & Post-partum Guidelines (mothers) **OR 22.1 (95%CI 8.0 to 61.4)** Adherence to Delivery & Post-partum Guidelines (newborns) **OR 24.1 (95%CI 8.1 to 72.0)** Adherence to Newborn Vaccination Guidelines **OR 4.7 (95%CI 1.2 to 18.3)**
Magge et al. 2015 [32]	705	Adherence to integrated management of childhood illness (IMCI) assessment; classification; treatment; counselling and communication; and coverage	Observation of nurse clinical practice by nurses with expertise in the integrated management of childhood illness	Adherence to Guidelines **OR 7.82 (95%CI 7.02 to 8.71)**
Martino et al. 2017 [24]	543	Motivational interviewing strategy adherence	Audio-tape review of counselling sessions and rating of adherence using the Independent Tape Rater Scale	Fundamental MI adherence N/S^a^ Advanced MI adherence N/S^a^
Pozen et al. 1976 [25]	300	Faculty member ratings of process of care	Medical record review	N/S^a^
Sox et al. 1998 [35]	3367	Adherence to emergency medicine guidelines for patients presenting to emergency	Medical record review	**64% vs. 55% mean compliance;** ***P*** **< 0.0001**
Wolfe et al. 2014 [27]	119	Achievement of CPR performance quality indicators: rate ≥ 100/min; depth ≥ 38 mm; cardiac compression fraction >90%; and ≤10% compressions with leaning (leaning greater than 2.5 kg).	Monitor and defibrillator with the capability to detect and record chest compressions and ventilations during resuscitation attempts	Rate ≥ 100/min **SMD .50 (95%CI 0.34 to 0.67)** Depth ≥ 38 mm **SMD 0.27 (95%CI 0.10 to 0.43)** Cardiac compression fraction >90% **SMD 0.43 (95%CI 0.26 to 0.59)** ≤ 10% compressions with leaning N/S^a^
Workneh et al. 2013 [33]	748	Adherence to clinical aspects of a comprehensive paediatric HIV visit as per national antiretroviral guidelines	Medical record review	Adherence to Guidelines **OR 2.70 (95%CI 2.39 to 3.04)**

bold text, *P* < .05

*N/S* non-significant

^a^insufficient data provided to calculate SMD/OR

### Patient experience

None of the three studies investigating the effect of clinical supervision of health professionals found a positive effect for patient satisfaction [25, 35, 38] (Table 3).

**Table 3:** Effect of supervision on patient experience

Study	N	Measure	Method	Results: Supervision vs. Control (OR >1 favours supervision)
Pozen et al. 1976 [25]	300	Patient satisfaction with outpatient medical service	Questionnaire	N/S^a^
Sox et al. 1998 [35]	1386	Patient satisfaction with the respect they received from staff; the completeness of care they had received; the explanations of their care; their waiting times; and the discharge instructions they received.	Follow-up telephone interview	OR 1.0 (95%CI 0.7 to 1.5)
White et al. 2010 [38]	170	Patient satisfaction with psychiatric care	Psychiatric Care Satisfaction Questionnaire (PCSQ)	N/S^a^

*N/S* non-significant

^a^insufficient data provided to calculate SMD/OR

## Discussion

Findings from 14 studies and 38,483 episodes of patient care indicate that clinical supervision of health professionals is associated with a significant improvement in the performance of some processes of care. This finding predominantly applies to improving processes in the performance of CPR through the application of reflective supervision; and medical/nursing care in African health settings through the application of supervision with a direct supervision component. The majority of studies that found a positive impact on process of care utilised a model of clinical supervision that included direct supervision. Studies that investigated a model of reflective supervision in the performance of process of care that also had access to real time feedback on clinician performance also found an improvement in process of care. Therefore, an accurate representation of clinical performance may be essential to improving process of care. The effects of clinical supervision on patient health outcomes and patient experience were investigated by six and three studies respectively, which was less than the 14 studies that investigated the impact of clinical supervision on performance of process of care. Clinical supervision of mental health professionals may be associated with a reduction in psychological symptoms for individuals with a mental health illness. Clinical supervision of health professionals in a small number of studies did not demonstrate any effect on patient experience.

Given our focus on the effectiveness of care, improvements in process measures are only meaningful if the process measure is associated with improved patient outcomes [12]. Ideally, clinical supervision should be utilised to produce a change in process of care, where the process has been demonstrated to produce improved patient health outcomes. For example, two studies included in this review implemented clinical supervision to enhance the practice of the integrated management of childhood illness guidelines, which have been shown to reduce mortality in African children under five years of age [29, 32, 39]. Both studies found a significant improvement in the process of care. Therefore, if the measures of process are indicative of compliance with evidence-based guidelines, improvement in practice will benefit patients. Health professionals have commonly reported that a lack of organisational support, resources and knowledge are three of the primary barriers to the uptake of evidence-based practice [40, 41]. Clinical supervision can address these barriers, providing clinicians with the support, resources and direction they require to enhance their uptake of evidence-based practice. Further research is required to investigate the effectiveness of clinical supervision as an implementation strategy compared to other strategies that have been shown to improve clinical performance, such as audit and feedback [42].

Direct supervision may be more useful in producing effective change in process of care than reflective supervision, as direct supervision allows for (1) greater levels of interaction between the supervisor and supervisee, and (2) a more accurate representation of clinical performance [43]. The majority of studies (*n* = 9) included in this review that found clinical supervision had a positive effect on process of care outcomes, utilised a model of clinical supervision where the supervisee’s clinical practice was directly supervised. However, three studies included in this review demonstrated that improvements in process of care can be achieved using a less direct model of clinical supervision, if accurate information on supervisees’ performance of care processes can be obtained via other means [26–28]. All three studies utilised an electronic device that provided real-time feedback on a clinician’s performance of CPR. Therefore, the decision whether to choose a direct or reflective model of supervision may depend on the clinical task being supervised and whether an accurate measure of clinician performance can be obtained without directly supervising clinical performance. When supervising a clinical task that cannot be accurately measured with an electronic device, direct supervision appears to be the most effective model to facilitate the provision of feedback, adapted to the supervisee’s needs, which is an important component of effective clinical supervision [44–46].

Another common attribute of supervision interventions that found positive effects for process of care is that the focus of supervision was to improve a clinical procedure/treatment technique (*n* = 4) or to improve practice in a specific area of practice (*n* = 7). Supervision of general practice could understandably be quite diverse and the focus of such supervision may be too broad to produce a change in health professional behaviour. In comparison, supervision that focuses on a particular standard of clinical practice allows both the supervisor and supervisee to direct their attention towards development of a skill set that will have an impact on clinical practice. Further research is required to establish the effect of supervision of general practice compared to supervision of a specific clinical area or procedure.

Similar to other forms of health professional education, such as conferences, workshops and rounds, clinical supervision has a greater effect on process of care than patient health outcomes [47, 48]. However, our review did find evidence to indicate that clinical supervision of mental health professionals may reduce psychological symptoms for patients diagnosed with a mental illness [22, 23]. Mental health professionals, including social workers, psychologists and specialist nurses, acquire the skills required to facilitate clinical supervision in their undergraduate and postgraduate studies, and have widely adopted the practice of clinical supervision and perceive clinical supervision as an effective tool for the development of their clinical practice [49, 50]. Therefore, mental health professionals possess the skills required to facilitate the development of fellow colleagues and the receptiveness required to utilise clinical supervision for their personal skill development.

Findings from this review have broadened our understanding of the effects of clinical supervision on quality of care. Clinical supervision has been found to be associated with improved medical resident adherence to guidelines in the inpatient setting [9]. Our results not only support this association but, through analysis of a further 13 studies, support the use of clinical supervision to improve process measures in nursing, allied health and medical professionals across both inpatient and community settings. Additionally, a lack of evidence to support a direct relationship between clinical supervision and patient health outcomes identifies an opportunity for further investigation. Lastly, this review mirrors the findings of a prior review that investigated the effects of clinical supervision on patient safety, by highlighting the importance of a direct supervision component in achieving changes in health professional behaviour that can impact on quality of care [8].

There are several limitations that need to be considered when interpreting the results of this review. First, only two studies [24, 37] investigating the effects of clinical supervision on process of care utilised a randomised controlled design. While it is difficult for studies investigating medical education to randomise participants, without adequate randomisation there is increased risk of bias in interpretation of the results [51]. However, MERSQI scores of the included studies averaged ≥11 indicative of a higher quality study, even without the randomised control. Second, most of the studies included in this review measured process of care by reviewing a patient medical record documentation. While this is a convenient method, the information obtained is only as accurate as the available documentation [12, 52]. Alternatively, process of care was measured by direct observation of patient care in two studies [29, 32]. This provides an accurate depiction of the process of care but also introduces the possibility of observer bias. Finally, due to the heterogeneity of the studies included in this review, there are several questions that still remain unanswered in regards to clinical supervision and its operationalisation. Specifically, this review included studies that investigated supervision of a wide range and experience of health professionals, who were supervised in the performance of clinical duties or procedures that were diverse across the studies. Therefore, it is still unknown 1) which health professional benefits most from clinical supervision and at what level of experience; 2) which clinical duties or procedures are most influenced by clinical supervision; and 3) who should provide the clinical supervision. Furthermore, a limitation of many of the included studies in this review was the lack of operationalisation of clinical supervision. Clearer descriptions of the participants, quantity and content of clinical supervision will enable health professionals to better determine the model of clinical supervision that is associated with improved quality of care.

## Conclusion

Clinical supervision of health professionals is associated with effectiveness of care. The review found significant improvement in the process of care that may improve compliance with processes that are associated with enhanced patient health outcomes. While few studies demonstrated a direct effect on patient health outcomes, clinical supervision of mental health professionals may be associated with a reduction in psychological symptoms of patients diagnosed with a mental illness. No association was found between clinical supervision of health professionals and the patient experience dimension of quality of care.

## Additional files

Additional file 1: Medline search strategy. Example of the search strategy used to search the Medline database. (DOCX 15 kb)

Additional file 2: Summary table of included studies. Table outlining the characteristics of studies included in this review [22–38]. (DOCX 45 kb)
