# Supplementary material for: Does clinical supervision of healthcare professionals improve effectiveness of care and patient experience? A systematic review
Source: BMC Health Serv Res. 2017 Nov 28;17:786. doi: 10.1186/s12913-017-2739-5 (PMC5706384; doi:10.1186/s12913-017-2739-5)
Supplement: Supplementary file 1 — Medline search strategy. Example of the search strategy used to search the Medline database. (DOCX 15 kb) [file 12913_2017_2739_MOESM1_ESM.docx]

**Additional file 1 Medline search strategy (n= 6311)**

S1. superv* or mentor* or debrief* or reflective practice

S2. patient outcomes or clinical outcomes or client outcomes or patient care or quality of care or patient experience or adherence or compliance

S3. S1 AND S2

S4. Limit S3 to humans

S5. Limit S4 to English language
