# Supplementary material for: Does clinical supervision of healthcare professionals improve effectiveness of care and patient experience? A systematic review
Source: BMC Health Serv Res. 2017 Nov 28;17:786. doi: 10.1186/s12913-017-2739-5 (PMC5706384; doi:10.1186/s12913-017-2739-5)
Supplement: Supplementary file 2 — Summary table of included studies. Table outlining the characteristics of studies included in this review [22–38]. (DOCX 45 kb) [file 12913_2017_2739_MOESM2_ESM.docx]

**Additional file 2 Summary table of included studies**

| **Study** | **Discipline** | **Study Design** | **Setting** | **Sample Size** | **Clinical Practice Supervised** | **Description of Supervision** | **Comparison** | **Study Quality (MERSQI)**  **(/18)** |
| --- | --- | --- | --- | --- | --- | --- | --- | --- |
| Anatole et al 2013 [29] | Nursing | Single group, pre-test post-test | 21 Health Centres, Rwanda | 2649 consultations | **Specified Area of Practice:** Nursing management of paediatric, adult and antenatal patients | Direct and reflective supervision of clinical management by a nurse with several years of experience and formal training in their clinical speciality  Frequency: supervision visits every 4-6 weeks | Historical control pre-supervision intervention | 12.2 |
| Bambling et al 2006 [22] | Social work, psychology & mental health | Randomised controlled trial | Community Centres, Australia | 103 clients | **Specified Area of Practice:** Problem solving treatment of clients with major depression | Reflective supervision (two different models) of junior mental health professionals by a mental health professional with two or more years’ experience as a supervisor  Frequency: 8 sessions; 1 pre-treatment, 7 post-treatment | Unsupervised therapists | 15.8 |
| Bradshaw et al 2007 [23] | Mental health nursing | Prospective cohort | Community and inpatient health services,  UK | 93 clients | **Specified Area of Practice:** Psychosocial intervention for clients diagnosed with psychosis | Reflective supervision in groups of two. Supervisors provided feedback on the nurses’ performance and discussed the application of psychosocial intervention with reference to their current caseload  Frequency: fortnightly supervision | Unsupervised mental health nurses | 11.5 |

| **Study** | **Discipline** | **Study Design** | **Setting** | **Sample Size** | **Clinical Practice Supervised** | **Description of Supervision** | **Comparison** | **Study Quality (MERSQI)**  **(/18)** |
| --- | --- | --- | --- | --- | --- | --- | --- | --- |
| Claridge et al 2011 [34] | Medical | Retrospective cohort | Trauma centre,  North America | 376 patients | **Specified Area of Practice:** Emergency management of patients presenting with a blunt splenic injury | Direct supervision of residents by an in-house surgical attending  Frequency: NR | Indirect supervision by an attending surgeon on call | 14 |
| Couper et al 2015 [26] | Medical and nursing | Prospective cohort | Three acute hospitals,  UK | 1395 cardiac arrest events | **Procedure / Treatment Technique:** CPR of patients with cardiac arrest | Reflective group supervision of the multi-disciplinary emergency team by senior medical professionals  Frequency: weekly supervision | 1) Audiovisual feedback without reflective group supervision  2) No reflective group supervision OR audiovisual feedback  3) Historical control pre-supervision intervention | 15 |
| Edelson et al 2008 [28] | Medical | Single group, pre-test post-test | Acute teaching hospital,  North America | 224 cardiac arrest events | **Procedure / Treatment Technique:** CPR of patients with cardiac arrest | Reflective group supervision of internal medicine residents by a senior medical professional  Frequency: weekly supervision | Historical control pre-supervision intervention | 12 |
| Fatti et al 2013[30] | Nursing | Single group, pre-test post-test | 31 public nurse-led antenatal and delivery health facilities,  South Africa | 27458 patients | **Specified Area of Practice:** Prevention of mother to child HIV transmission | Direct and reflective supervision of nurses by experienced nurse mentors with knowledge of processes to prevent mother to child transmission of HIV  Frequency: fortnightly supervision | Pre-supervision intervention control group | 13.1 |
| **Study** | **Discipline** | **Study Design** | **Setting** | **Sample Size** | **Clinical Practice Supervised** | **Description of Supervision** | **Comparison** | **Study Quality (MERSQI)**  **(/18)** |
| Green et al 2014 [31] | Nursing | Single group, pre-test post-test | Eight medical centres,  South Africa | 160 medical records | **Specified Area of Practice:** Antiretroviral therapy for HIV positive adults | Direct supervision of nurses by a nurse experienced in the administration of antiretroviral therapy  Frequency: 40 hours of supervision | Pre-supervision intervention control group | 12 |
| Gupta et al 2016 [36] | Medical, Nursing and allied health | Single group, pre-test post-test | Seven primary care centres, Canada | 384 spirometry tests | **Procedure / Treatment Technique:** Spirometry | Direct and reflective supervision of physicians, nurses and respiratory therapists by a physician/certified respiratory educator with spirometry expertise  Frequency: NR | Pre-supervision intervention control group | 13 |
| Jayanna et al 2016 [37] | Nursing | Cluster randomised controlled trial | 108 primary health centres, India | 1078  Medical records | **Specified Area of Practice:** Intra-partum and post-partum care | Direct and reflective supervision of nurses by a nurse trained in midwifery  Frequency: one supervision visit every 2 months | Unsupervised nurses | 15 |
| Magge et al 2015 [32] | Nursing | Single group, pre-test post-test | 21 health centres, Rwanda | 705 consultations | **Specified Area of Practice:** Integrated management of acute childhood illness | Direct and reflective supervision of nurses by a nurse trained in integrated management of childhood illness  Frequency: one supervision visit every 4-6 weeks | Pre-supervision intervention control group | 13.1 |

| **Study** | **Discipline** | **Study Design** | **Setting** | **Sample Size** | **Clinical Practice Supervised** | **Description of Supervision** | **Comparison** | **Study Quality (MERSQI)**  **(/18)** |
| --- | --- | --- | --- | --- | --- | --- | --- | --- |
| Martino et al 2016 [24] | Allied Health | Randomised controlled trial | 11 outpatient community addiction treatment programs | 385 patients  543 consultations | **Procedure / Treatment Technique:** Motivational interviewing to treat substance abuse | Reflective supervision of counsellors by an experienced counsellor  Frequency: one supervision session after consultation of each new patient  (average of six per clinician) | Usual supervision practice control group  Frequency: no minimum requirements  (average of less than one per clinician) | 16 |
| Pozen et al 1976 [25] | Medical | Prospective cohort | Six outpatient clinics,  North America | 300 patients | **General Practice:** Outpatient medical assessment and treatment | Direct supervision of house officers by faculty staff members.  Frequency: weekly supervision | 1) Administration support and faculty staff on call  2) Faculty staff on call | 12 |
| Sox et al  1996 [35] | Medical | Retrospective cohort | Five acute teaching hospitals,  North America | 3367 patients | **General Practice:** Emergency department medical management of patients | Direct supervision of residents, in the emergency department setting at the time of patient management, by an attending physician  Frequency: NR | Episodes of care where the resident was not directly supervised | 13.5 |
| White et al 2010 [38] | Mental health nursing | Prospective cohort | 17 inpatient and community mental health facilities,  Australia | 170 patients | **General Practice:** Mental health nursing care | Reflective group supervision of mental health nurses by nurses trained to provide supervision  Frequency: NR | Unsupervised mental health nurses | 10 |
| Wolfe et al 2014 [27] | Medical and nursing | Single group, pre-test post-test | Paediatric ICU,  North America | 119 patients | **Procedure / Treatment Technique:** Paediatric CPR | Reflective group supervision of the arrest team led by critical care fellows  Frequency: Following every CPR event | Pre-supervision intervention control group | 12.5 |
| **Study** | **Discipline** | **Study Design** | **Setting** | **Sample Size** | **Clinical Practice Supervised** | **Description of Supervision** | **Comparison** | **Study Quality (MERSQI)**  **(/18)** |
| Workneh et al 2013 [33] | Medical and nursing | Single group, pre-test post-test | Four clinical outreach clinics,  Botswana | 374 patients | **Specified Area of Practice:** Antiretroviral therapy and monitoring of paediatric HIV patients | Direct supervision of medical officers and nurses by a senior medical officer and/or nurse who are experienced in the management of paediatric patients with HIV  Frequency: monthly supervision | Pre-supervision intervention control group | 12 |

NR: Not reported
